# Prevalence and significance of serum 14–3-3η in juvenile idiopathic arthritis

**DOI:** 10.1186/s12969-021-00502-8

**Published:** 2021-02-16

**Authors:** Iris Reyhan, Olga S. Zhukov, Robert J. Lagier, Robert F. Bridgforth, Gary J. Williams, Joanna M. Popov, Stanley J. Naides, Andreas Reiff

**Affiliations:** 1 University of Southern California-Children’s Hospital of Los Angeles, 4650 Sunset Blvd. Mailstop #60, Los Angeles, CA 90027 USA; 2 33608 Ortega Highway, San Juan Capistrano, CA 92675 USA; 3 33072 Sunharbor, Dana Point, CA 92629 USA

**Keywords:** Juvenile idiopathic arthritis, CCP antibody, Rheumatoid factor, Biomarker, 14–3-3η(eta)

## Abstract

**Background:**

Prompt diagnosis of juvenile idiopathic arthritis (JIA) is important to avoid long term complications. Elevated serum 14–3-3η levels improve the diagnostic sensitivity of rheumatoid factor (RF) and cyclic citrullinated peptide (CCP) antibody in adult rheumatoid arthritis (RA), and have been associated with more severe phenotype. We investigated the prevalence and clinical significance of serum 14–3-3η in different types of JIA.

**Methods:**

JIA patients (*n* = 151) followed by the Pediatric Rheumatology Core at Children’s Hospital of Los Angeles were categorized into 5 groups: polyarticular JIA RF+ (PJIA RF+; *n* = 39), PJIA RF- (n = 39), psoriatic arthritis (PsA; *n* = 19), enthesitis-related arthritis (ERA; *n* = 18), and oligoarticular JIA (OJIA [control group]; *n* = 36). RF, CCP antibody, and 14–3-3η were measured for all patients. 14–3-3η serum levels > 0.2 ng/mL were considered positive. Disease activity was assessed by the Juvenile Arthritis Disease Activity Score-71 (JADAS-71).

**Results:**

Elevated 14–3-3η levels were detected in 34/151 (23%) patients, and across all groups tested. Most patients with 14–3-3η had titers ≥4 times above the cutoff value. The majority (22, 65%) of 14–3-3η-positive patients were also positive for RF or CCP antibodies, 16 (47%) were positive for all 3, and 12 (35%) were single-positive for 14–3-3η. The highest prevalence of 14–3-3η was in PJIA RF+ patients (49%), followed by OJIA (22%). Positivity for 14–3-3η was not significantly associated with disease activity or age at diagnosis.

**Conclusion:**

Serum 14–3-3η can be detected in all forms of JIA tested but appears to be most common in PJIA RF+. 14–3-3η does not appear to correlate with disease activity in JIA.

## Background

Juvenile idiopathic arthritis (JIA) is the most common pediatric rheumatologic disease that may persist into adulthood and impact quality of life. The diagnosis is mainly based on clinical assessment rather than relying on laboratory testing. The disease course and prognosis of JIA may vary based on multiple factors such as presence of the biomarkers rheumatoid factor (RF) and cyclic citrullinated peptide (CCP) antibodies [1–4]. RF-seropositive polyarticular JIA (PJIA RF+) has similar features as adult rheumatoid arthritis (RA) and may extend into adulthood. Thus, PJIA RF+ patients are more susceptible to developing severe disease and are at higher risk of erosive joint damage [1, 5, 6]. Based on a 17-year cohort study, outcomes are best predicted at 5-year follow up, since there are no specific diagnostic markers for JIA [7].

The biomarker 14–3-3η is highly sensitive and specific for RA in adults. 14–3-3η is a protein that activates pathways associated with the production of proinflammatory cytokines [8, 9]. There are 7 isoforms of the 14–3-3 protein. Only 14–3-3η is found in the serum and synovial fluid in patients with RA, but at a 3–5 fold higher concentration in synovial fluid compared to matched serum, consistent with intra-articular production [9, 10]. Elevated serum 14–3-3η levels improve the diagnostic sensitivity of RF and CCP antibody in adult RA and are associated with a more severe RA phenotype [9]. The combination of the 3 serum markers (presence of 14–3-3η, RF, and/or CCP antibodies) has been reported to increase the diagnostic sensitivity to 78% for early RA and 96% for established RA [8, 9]. Based on previous studies serum 14–3-3η has enhanced the detection of early-RA by 32 and 22% over RF and CCP antibody, respectively [9].

Serum 14–3-3η titer may also have prognostic utility. A few studies have reported an association of elevated 14–3-3η with development of RA in patients with arthralgia [11–13]. In addition, patients with early RA and detectable 14–3-3η at baseline had more erosive disease at 5 years compared to those with normal 14–3-3η levels at baseline [12]. Further, patients with adult RA in apparent clinical remission after 18 months of therapy had worse Sharp scores at 30 months if their 18-month 14–3-3η level was > 0.5 ng/mL [14]. Additionally, PsA patients have been reported to have more erosive disease at a lower level of 14–3-3η compared to RA patients [9].

The prevalence and sensitivity of this biomarker have not been studied in a large pediatric population with JIA, except for three abstract publications of two pediatric JIA populations [15–17]. This study reports the prevalence and clinical significance of serum 14–3-3η in a large population of children with various subtypes of JIA.

## Materials and methods

### Patient cohort

Patients for this study were recruited from the patient population examined and followed by the Pediatric Rheumatology Core at Children’s Hospital of Los Angeles (PRC-CHLA). Patients with a diagnosis of OJIA (persistent OJIA and extended OJIA), PJIA RF+, PJIA RF-, ERA, and PsA were enrolled. Persistent OJIA is defined as arthritis affecting up to 4 joints during the first 6 months of disease course vs. extended OJIA is defined as progression of the disease to greater than 4 joints after the first 6 months of disease. Subjects met the diagnostic International League of Associations for Rheumatology (ILAR) criteria of OJIA, PJIA RF+, PJIA RF-, and ERA. Since this study was solely to assess this new biomarker among JIA population, children with a history of other autoimmune disorders or other forms of arthritis were excluded from the study. OJIA group was chosen as control group, as it was hypothesized, OJIA group would not have elevated 14–3-3η, as the course of disease for OJIA is different compared to other JIA subtypes. The protocol was reviewed and approved by the Children’s Hospital of Los Angeles institutional review board. Consent and assent were obtained from all subjects in order to participate in the study.

A one-time blood draw (1 red top tube ~ 5 mL) for 14–3-3η via venipuncture was obtained during a routine laboratory and rheumatology visit, or scheduled infusion, from 151 patients: 39 with PJIA RF+, 39 with PJIA RF-, 36 with OJIA (32 persistent OJIA, 4 extended OJIA), 18 with ERA, and 19 with PsA. Other laboratory studies, including erythrocyte sedimentation rate (ESR) and C-reactive protein (CRP), were obtained at the same time. Specimens for CCP antibody, RF, antinuclear antibody (ANA), and HLA-B27 testing were obtained at the same time as well, if the values were missing in their chart (paper and/or electronic).

### Analyses of serum 14–3-3η

Samples for 14–3-3η were centrifuged and the serum was collected. Serum was stored in a freezer at CHLA. Then the batch was packaged on dry ice and mailed to Quest Diagnostics Nichols Institute overnight. 14–3-3η was measured via ELISA at Quest Diagnostics [2]. A 14–3-3η serum level of > 0.2 ng/mL was considered positive. This titer level was validated as appropriate level based on previous adult studies and titer level used by laboratories. Fisher’s exact test was used to assess differences in prevalence of positive 14–3-3η between the OJIA (control) group and all other groups. The Cochran-Armitage trend test was used to evaluate the linear association of positive 14–3-3η with composite RF and CCP antibody positivity. Fisher’s exact test was also used to assess the association of 14–3-3η positivity with ANA and CRP.

### Disease activity assessment

Disease activity was assessed with the JADAS-71 and Childhood Health Assessment Questionnaire (CHAQ) [18, 19]. All statistical analyses were performed in R [20]. The linear association of positive 14–3-3η and disease activity was assessed via Cochran-Armitage Trend Test within each JIA group. Furthermore, positive 14–3-3η was assessed among patients based on treatment exposures: nonsteroidal anti-inflammatory drugs (NSAID), disease-modifying antirheumatic drugs (DMARDs) such as methotrexate, and biologics such as anti-tumor necrosis factor α (anti-TNFα), tocilizumab, abatacept, tofacitinib, ustekinumab, secukinumab, and rituximab.

### Serum 14–3-3η vs. age of onset and age at the time of blood draw

Differences in age at time of diagnosis as well as age at time of blood draw for 14–3-3η positive and negative subjects were assessed with Kruskal-Wallis tests. All other comorbidities and medications were obtained from the patient chart.

## Results

**Fig. 1:**
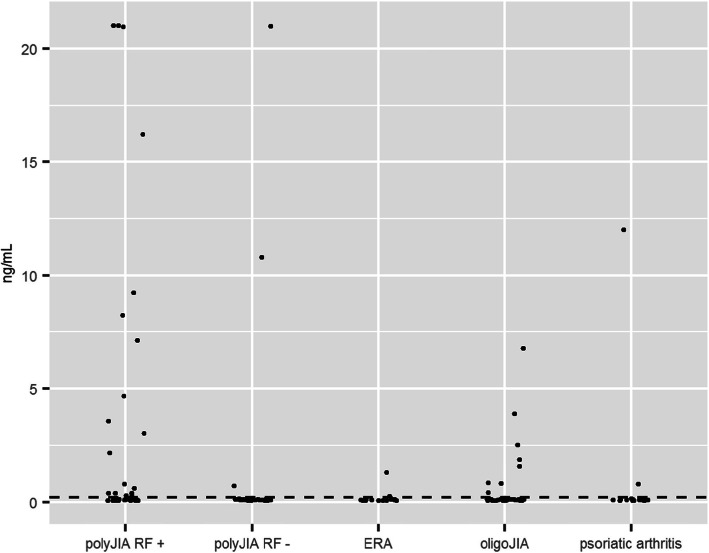
14-3-3η level within different JIA groups

Serum 14–3-3η was detected at levels above the 0.2 ng/mL cutoff among all JIA subtypes (23%, 34/151), including the OJIA group. As demonstrated in Table 1 and Fig. 1, PJIA RF+ group had the highest rate of positive 14–3-3η at (49%; 19/39). The PJIA RF- group had the lowest rate of positive 14–3-3η (8%, 3/39). Remarkably, the OJIA group had the second highest rate of positive 14–3-3η. There were 4 patients with extended OJIA within OJIA group and only 1 patient had positive 14–3-3η. Nevertheless, the odds of a positive 14–3-3η tests in PJIA RF+ subjects was 3.3 times that of OJIA subjects (OR 3.27, *p*-value 0.029). Similarly, the odds of a positive 14–3-3η tests was nearly 8 fold greater in PJIA RF+ subjects than in PsA subjects (OR 7.8, p-value 0.008).

**Table 1:** Two group comparison of 14–3-3η test results

Test Group	14–3-3η+	95% CI for Positive Proportion	OR	95% CI for OR	p-Value
**PJIA RF+**	19/39 (49%)	32 to 65%	3.27^a^	1.10 to 10.49	**0.029**
			7.81^b^	1.52 to 78.8	**0.008**
**PJIA RF-**	3/39 (8%)	2 to 21%	0.30^a^	0.05 to 1.38	0.105
			0.71^b^	0.07 to 9.28	1.000
**ERA**	2/18 (11%)	1 to 35%	0.44^a^	0.04 to 2.62	0.466
			1.06^b^	0.07 to 16.3	1.000
**OJIA**	8/36 (22%)	10 to 39%	2.39^b^	0.41 to 25.8	0.465
**PsA**	2/19 (11%)	1 to 33%			

*CI* Confidence interval. OR = Odd’s Ratio for 14–3-3η positive result for total population compared to control group (OJIA^a^ or PsA^b^)

### Gender comparison of serum 14–3-3η results

Among the 5 subtypes of JIA subjects included in this cohort, 78% (118/151) were female. Fifty-seven of patients within this cohort were within the PJIA RF+ and PJIA RF- groups, which also had the highest female to male ratios (87% female in both groups). Positive 14–3-3η results were more common among female patients (31/118; 26%) than male patients (3/33; 9%). The highest rate of 14–3-3η positivity among female group was in the PJIA RF+ female group (17/34; 50%). The second highest prevalence of positive 14–3-3η among female subjects was among OJIA (8/28; 29%) compared to other groups: PsA (2/11; 18%), ERA (1/11; 9%) and PJIA RF- (3/34; 9%). None of the male patients in the PJIA RF-, OJIA, or PsA groups had a positive 14–3-3η titer (PJIA RF+ 2/5; 40% and ERA 1/7; 14%).

### Comparison of 14–3-3η, RF, and CCP antibodies within each group

OJIA subjects had the highest rate of positive 14–3-3η (19%) in the absence of other biomarkers: RF or CCP antibody (Table 2). Of note, 8 out of 36 (22%) of the OJIA patients had detectable 14–3-3η, most of whom (88%) were positive for only 14–3-3η (negative for RF and CCP antibodies; Table 2). Also, 4 out of 36 OJIA patients had extended OJIA, while only one out of 4 of these patients had positive14–3-3η. The OJIA, PsA, and ERA groups were the least likely to be positive for 14–3-3η, in addition to RF or CCP antibody or both. Overall, the PJIA RF+ group had a higher proportion of triple positive patients with 14–3-3η, CCP antibody, and RF (41%) than any other group.

**Table 2:** Comparison of 3 biomarkers: RF, CCP antibodies, and 14–3-3η

Biomarker	PJIA RF+ n = 39	PJIA RF- n = 39	ERA n = 18	PsA n = 19	OJIA n = 36	Total n = 151
Positive 14–3-3η	19 (49%)	3 (8%)	2 (11%)	2 (11%)	8 (22%)	34 (23%)
Positive 14–3-3η without RF, CCP	0 (0%)	1 (3%)	2 (11%)	2 (11%)	7 (19%)	12 (8%)
Positive CCP	30 (77%)	4 (10%)	0 (0%)	0 (0%)	2 (6%)	36 (24%)
Positive 14–3-3η, CCP	16 (41%)	2 (5%)	0 (0%)	0 (0%)	1 (3%)	19 (13%)
Positive RF	39 (100%)	0 (0%)	0 (0%)	0 (0%)	0 (0%)	39 (26%)
Positive 14–3-3η, RF	19 (49%)	0 (0%)	0 (0%)	0 (0%)	0 (0%)	19 (13%)
Positive 14–3-3η, RF, CCP	16 (41%)	0 (0%)	0 (0%)	0 (0%)	0 (0%)	16 (11%)^#^
Positive ANA	20 (51%)	15 (38%)	10 (56%)	10 (56%)	23 (64%)	78 (52%)
Positive HLA-B27	2/38 (5%)^a^	2/37 (5%)^a^	6 (33%)	2 (11%)	2 (6%)	14/148 (9%)^a^

^a^Number of patients with HLA-B27. Both PJIA RF- and PJIA RF+ groups were each missing HLA-B27 results for 3 patients

^#^Note: *P*-value < 0.0001 for trend in positivity across all 3 biomarkers

### Comparison of RF and CCP antibodies with 14–3-3η

There was a linear trend in 14–3-3η positivity with cumulative RF/CCP antibody positivity. Of the 30 subjects positive for both RF and CCP antibody, 16 (53% [34, 72%, respectively]) were positive for 14–3-3η. Among the 15 subjects positive for either RF or CCP antibody, 6 (40% [16, 68%]) were positive for 14–3-3η. Lastly, among the 106 subjects negative for both RF and CCP antibody, only 12 (11% [6, 19%]) were positive for 14–3-3η (*p*-value for trend in proportions = 3.5 × 10^− 7^). RF positivity was strongly associated with CCP antibody positivity (*p* < 2.2 × 10^− 16^). In PJIA RF+ patients 77% (30/39) had a positive CCP antibody (Table 2), while 41% (16/39) were positive for RF, CCP antibody and 14–3-3η.

Positive CCP antibody levels were more commonly observed in PJIA groups. However, OJIA, PsA, and ERA groups were more likely to have a positive 14–3-3η compared to CCP antibody. Twenty two percent of OJIA subjects had a detectable 14–3-3η level, while only 6% had a positive CCP antibody. However, PJIA RF+ group had a higher prevalence of CCP antibody (77%) vs. 14–3-3η (49%).

### Comparison of serum 14–3-3η at different cut off levels of > 0.4 ng/ml and > 0.8 ng/ml

Overall, a similar proportion of patients with elevated 14–3-3η titer > 0.2 ng/ml had 14–3-3η level equal to or greater than 0.4 ng/ml and 0.8 ng/ml. There was a wide range of 14–3-3η titer, from 0.2 ng/ml to > 20 ng/ml, among all groups. As mentioned previously, 23% of all patients had a positive 14–3-3η result at > 0.2 ng/ml. Eighty eight percent (30/34) of those patients had 14–3-3η titers of > 0.4 ng/ml, and 71% (24/34) had titers > 0.8 ng/ml. Among OJIA, PJIA RF-, and PsA groups, all subjects with positive 14–3-3η had 14–3-3η titers at 0.4 ng/ml or greater. However, large numbers (84%, 16/19) of PJIA RF+ patients also had 14–3-3η levels > 0.4 ng/ml. Additionally, 58% of PJIA RF+ (11/19) and 67% of PJIA RF- (2/3) patients with positive14–3-3η had titers 10 times (> 2 ng/ml) greater than the cutoff value. Only 3 (38%) OJIA, 1 (50%) PsA, and none of ERA patients had elevated 14–3-3η titers 10 times higher than the baseline.

### Comparison of 14–3-3η vs. ANA and HLA-B27

Positive ANA was found among all groups; the highest prevalence was among OJIA subjects (64%) and the lowest prevalence was among PJIA RF- (38%). Thirty-one percent of PJIA RF+ population had a positive ANA and 14–3-3η compared to OJIA 19%, PsA 11%, ERA 6%, and PJIA RF- 0% (Table 2). Overall, 60% of PJIA RF+ patients with positive ANA had serum 14–3-3η levels above baseline. There was no association between positive ANA and 14–3-3η among all groups (*p*-value = 0.119, OR 1.96).

As expected, positive HLA-B27 was found at a higher rate among ERA patients 33%, vs. PsA 11%, OJIA 6%, PJIA RF+ 5%, and PJIA RF- 5%. There was no correlation between positive 14–3-3η and elevated CRP (p-value = 1, OR 1.1).

### Comparison of 14–3-3η and disease activity

There was no association between disease activity based on JADAS-71 and positive 14–3-3η results, in any of the JIA types (Table 3). There was no association between age of onset, age at the time of blood draw, and having a positive 14–3-3η titer. Furthermore, no correlations between detectable 14–3-3η level and treatments (NSAIDs, DMARD, or biologics) were observed (Table 4). Methotrexate and anti-TNFα were the most common therapies used. Interestingly, a large proportion of OJIA patients required DMARD (81%) and/or anti-TNFα (42%) therapy. OJIA patients required biologics less commonly compared to the other groups. All of the OJIA subjects with positive 14–3-3η titer required DMARDs, 38% of whom also required biologics. However, all of PJIA RF- subjects with elevated 14–3-3η level required biologics, which was different than PJIA RF+ (84%), PsA (50%), and ERA (50%).

**Table 3:** JIA Disease Activity vs. 14–3-3η

Disease activity	# Positive 14–3-3η	Total #	p-Value
**OJIA**			
Inactive	6 (30%)	20	0.59
Mild	0 (0%)	7	
Moderate	1 (20%)	5	
Severe	1 (25%)	4	
**PJIA RF-**			
Inactive	0 (0%)	10	0.81
Mild	2 (20%)	10	
Moderate	0 (0%)	8	
Severe	1 (9%)	11	
**PJIA RF+**			
Inactive	2 (25%)	8	0.59
Mild	8 (67%)	12	
Moderate	5 (45%)	11	
Severe	4 (50%)	8	
**PsA**			
Inactive	0 (0%)	6	0.61
Mild	1 (17%)	6	
Moderate	1 (20%)	5	
Severe	0 (0%)	2	
**ERA**			
Inactive	1 (11%)	9	1
Mild	0 (0%)	2	
Moderate	1 (20%)	5	
Severe	0 (0%)	2

**Table 4:** Drug exposure in each group, 14–3-3η

JIA Type	NSAIDs		DMARDs		Biologics	
	14–3-3η+	14–3-3η-	14–3-3η+	14–3-3η-	14–3-3η+	14–3-3η-
PJIA RF+	16/19 (84%)	19/20 (95%)	17/19 (89%)	19/20 (95%)	16/19 (84%)	17/20 (85%)
PJIA RF-	1/3 (33%)	30/36 (83%)	2/3 (67%)	33/36 (92%)	3/3 (100%)	31/36 (86%)
ERA	0/2 (0%)	11/16 (69%)	0 /2 (0%)	11/16 (69%)	1/2 (50%)	14/16 (88%)
PsA	2/2 (100%)	10/17 (59%)	1/2 (50%)	13/17 (76%)	1/2 (50%)	14/17 (82%)
OJIA	6/8 (75%)	21/28 (75%)	8 /8 (100%)	21/28 (75%)	3/8 (38%)	12/28 (43%)

### Comorbidities among OJIA and serum 14–3-3η level

Overall, uveitis was more common among OJIA patients than in other groups. Chronic uveitis was found in 12 (33%) of 36 OJIA patients, more frequently than in PJIA RF+ (0/39), PJIA RF- (6/39; 15%); PsA (3/19; 16%), and ERA (5/18; 28%). Among OJIA patients with positive 14–3-3η titer, only 3 (38%) had a diagnosis of uveitis. Although the numbers were too small for statistical analysis, uveitis was as common in OJIA patients with 14–3-3η (9/28; 33%) as in those without 14–3-3η (3/8; 38%). In other JIA groups, 14–3-3η was not detected in patients with uveitis.

## Discussion

To date this is the largest published pediatric study assessing the importance of 14–3-3η in children with JIA. Overall, in our study, the prevalence of JIA was much higher among female patients (78%), compared to previous reports of a 2 to 1 female to male ratio, respectively [1, 6, 21]. PJIA RF+ mimics adult RA and, as expected, the PJIA RF+ population had the highest prevalence of 14–3-3η positivity (49%). There was a positive correlation with elevated serum 14–3-3η titer among PJIA RF+ group compared to OJIA controlled group (*p* value = 0.03) and PsA (p value = 0.008). The prevalence of 14–3-3η positivity in children with PJIA RF+ was similar to that in another study on early undifferentiated polyarthritis in adults. However, in both early and established RA, a higher prevalence of 14–3-3η (68%) was reported compared to our pediatric population [21, 22]. In a previous report, 14–3-3η was positive in 21% of patients with early RA who were seronegative for RF and CCP antibodies, and in 67% of patients with seronegative established RA and in a pediatric study, 31% of PJIA RF- patients had positive 14–3-3η levels [16, 23]. This differs from our results, which showed a much lower frequency (8%) in the PJIA RF- group.

To date there are only two pediatric 14–3-3η studies on JIA, which were published as conference abstracts. One of these studies included a much smaller population of JIA patients. Similar to our study, the highest prevalence of positive 14–3-3η level was seen among PJIA RF+ patients [15–17]. There were some differences between these reports: Rosenberg et al. study described no significant association between positive RF and 14–3-3η titer in JIA, yet a correlation between positive 14–3-3η and positive RF and CCP antibodies in PJIA was observed in Feller/Dalrymple et al. study [15, 16]. We found a positive correlation between 14 and 3-3η biomarker, RF, and CCP antibodies (*p*-value < 0.0001) in all groups. Serum 14–3-3η was found in all groups, including ERA subjects (11%). This finding differs from the Rosenberg et al. study, which reported no detectable 14–3-3η among their 4 ERA patients [17]. Of note, only one other pediatric study presented as an abstract evaluated 14–3-3η protein among children with JIA (including PJIA RF+, PJIA RF-, systemic onset JIA, systemic lupus erythematosus, and adults with RA), which included healthy children as controlled group and systemic lupus erythematosus (SLE) as disease control group. Based on this study elevated 14–3-3η titer were noted among all groups including the control group with higher titers among PJIA RF+, PJIA RF-, and adult RA [16]. The age group of these patients was not provided [16].

CCP antibody was more commonly observed in the PJIA RF+ group, similar to prior reports [24–26]. In our study, all groups except the ERA population had a positive CCP antibody, yet at a much lower frequency when compared to the PJIA RF+ group. There is a large variance in the reported prevalence of CCP antibody among different studies, with a positive association between RF and CCP antibodies [27, 28].

As expected, positive ANA serology was most commonly seen among the OJIA population [5]. Overall, there was a high prevalence of positive ANA among all groups, but to a lesser degree in PJIA RF- patients. However, in the presence of positive 14–3-3η titer, PJIA RF+ patients had the highest prevalence of positive ANA. No PJIA RF- patients had a positive 14–3-3 positive in the presence of a positive ANA result. Based on our search, there are no studies comparing the prevalence of positive ANA and 14–3-3η. Our study did not find any association between positive ANA and 14–3-3η.

In RA, 14–3-3η analysis has enhanced the detection of early-RA over RF and CCP antibodies, and it is associated with worse disease [9, 12]. Serum 14–3-3η was found to be a predictive biomarker to determine evolving RA and radiographic changes in early RA [11, 12]. Even though elevated 14–3-3η was reported to be associated with radiographic damage and disease progression in adults with established RA, no significant association between positive 14–3-3η and Disease Activity Score – 28 (DAS28) was reported [10, 23]. In our cohort, we also did not note any association between positive 14–3-3η and JADAS-71 in children with JIA [10]. A prior adult study, in abstract form, reported that 14–3-3η levels were modifiable after treatment with adalimumab and were predictive of treatment response in adults with PsA and RA [29]. Nevertheless, another study revealed no difference in pretreatment and posttreatment 14–3-3η levels in in RA patients treated with adalimumab, tofacitinib, or methotrexate [30]. Conversely, tocilizumab pretreatment 14–3-3η levels were predictive of remission and corresponded to DAS28 after therapy [30]. We found no correlation between positive 14–3-3η titer and administered therapy: NSAIDs vs. DMARDs vs. biologics. Many patients had elevated 14–3-3η titers even with exposure to biologics. Both OJIA and PJIA RF- patients with positive 14–3-3η were treated with DMARDs and biologics, respectively. Still, a high proportion (89%) of PJIA RF+ group with positive 14–3-3η required biologics.

Overall, serum 14–3-3η titer in 20% of all patients was twofold (> 0.4 ng/ml) higher than the cutoff level (> 0.2 ng/ml). Most patients with a positive 14–3-3η had titers 4 times greater than this cutoff value. Surprisingly, although 88% of 14–3-3η-positive OJIA patients had serum 14–3-3η titers 4 times higher than the cut off value, this group of patients had a lower proportion of patients (38%) with 14–3-3η titers 10 times higher than the baseline, especially compared to PJIA RF+ and PJIA RF- groups. Dalrymple et al. study had similar findings of positive 14–3-3η levels (> 0.5 ng/mL) among PJIA RF+, PJIA RF-, and OJIA [16]. However, lower prevalence of higher 14–3-3η titer (> 0.5 ng/mL) was noted among RA, systemic JIA, systemic lupus erythematosus, and healthy control groups [16].

OJIA patients were the only group with positive 14–3-3η and chronic uveitis. Furthermore, no correlations between 14 and 3-3η titer and disease activity, age of onset or age of blood draw, were observed.

## Conclusion

Although 14–3-3η titer is present among all types of JIA group, it may be a good prognostic factor among PJIA RF+. Therefore, further study is essential to evaluate the presence of this marker prior to diagnosis among patients with arthralgia and at the time of diagnosis with longer course of follow up.

### Limitations

Patients with new onset of JIA and established diagnosis were not differentiated. OJIA patients were used as a control group, yet there was a high prevalence of positive 14–3-3η among this group. Neither healthy subjects nor healthy children with arthralgia without evidence of arthritis were included in this study.

### Future studies

A prospective study of patients at onset of disease and follow up would be beneficial in determining the role of 14–3-3η biomarker. Comparing 14–3-3η level among JIA vs. patients with arthralgia, and healthy patients with follow up over several years would be informative in the role of this biomarker in disease classification. Additionally, measurement of 14–3-3η pretreatment and posttreatment, as well as comparison via imaging may be helpful to assess the value of 14–3-3η as a marker of joint damage and treatment response.

## Data Availability

The dataset used and analyzed for this study is available upon request.

## Abbreviations

ANA: Antinuclear antibody
anti-TNFα: Anti-tumor necrosis factor α
CHAQ: Childhood Health Assessment Questionnaire
CRP: C-reactive protein
CCP: Cyclic citrullinated peptide antibody
DAS-28: Disease Activity Score-28
DMARDs: Disease-modifying antirheumatic drugs
ERA: Enthesitis-related arthritis
ESR: Erythrocyte sedimentation rate
ILAR: International League of Associations for Rheumatology
IRB: Institutional review board
JIA: Juvenile idiopathic arthritis
JADAS-71: Juvenile Arthritis Disease Activity Score-71
NSAID: Nonsteroidal anti-inflammatory drugs
OJIA: Oligoarticular JIA
PJIA RF+: Polyarticular JIA RF+
PJIA RF-: Polyarticular JIA RF-
PsA: Psoriatic arthritis
RF: Rheumatoid factor antibody
RA: Rheumatoid arthritis
14–3-3η: 14–3-3η(eta)
